# Nonvitamin K antagonist oral anticoagulant activity: challenges in measurement and reversal

**DOI:** 10.1186/s13054-016-1422-2

**Published:** 2016-09-23

**Authors:** Karen S. Brown, Hamim Zahir, Michael A. Grosso, Hans J. Lanz, Michele F. Mercuri, Jerrold H. Levy

**Affiliations:** 1 Daiichi Sankyo Pharma Development, Edison, NJ USA; 2 Cardiothoracic Anesthesia and Critical Care, Duke University Medical Center, 2301 Erwin Rd., 5691H HAFS, Box 3094, Durham, NC 27710 USA

## Abstract

**Background:**

Four nonvitamin K antagonist oral anticoagulants (NOACs) are approved for the prevention of stroke in patients with nonvalvular atrial fibrillation and for the treatment of venous thromboembolism. These include the direct thrombin inhibitor dabigatran and the direct factor Xa inhibitors rivaroxaban, apixaban, and edoxaban. Bleeding is a complication for all anticoagulants and concerns regarding bleeding risk and the suitability of effective reversal strategies may be a barrier to their prescription. Despite the reduced risk of bleeding compared with vitamin K antagonists, questions persist regarding the management of bleeding related to NOAC use.

**Main text:**

To date, although a number of assays are responsive to NOACs, no single routine laboratory test has been identified to accurately measure the clinical anticoagulation state of patients on NOACs or established as a reliable predictor of bleeding risk. In addition, the establishment of a reliable human bleeding model to test novel inhibitors of the coagulation cascade has proved challenging. Although routine monitoring of anticoagulant levels is not necessary in patients taking NOACs, anticoagulant reversal and a means of measuring reversal may be required for patients who present with bleeding or require urgent surgery. Prothrombin complex concentrates are pooled plasma products containing varying amounts of inactive vitamin K-dependent clotting factors in addition to vitamin K-dependent proteins and can replenish factors in the intrinsic and extrinsic coagulation cascade, reversing an anticoagulant effect. Only one agent, idarucizumab, has been approved for rapid reversal of dabigatran-induced anticoagulation and one more agent, andexanet alfa, has been submitted for approval to reverse the anticoagulatory effects of direct and indirect factor Xa inhibitors.

**Conclusions:**

This review discusses the laboratory tests available for assessing anticoagulation, human models of bleeding, and the use of current strategies—including prothrombin complex concentrates for reversal of anticoagulation by NOACs—to manage bleeding in patients.

## Background

Four nonvitamin K antagonist oral anticoagulants (NOACs) are approved for the prevention of stroke in patients with nonvalvular atrial fibrillation (AF) and for the treatment of venous thromboembolism. These include the direct thrombin inhibitor dabigatran and the direct factor Xa (FXa) inhibitors rivaroxaban, apixaban, and edoxaban [1–4]. In phase 3 clinical testing, all four NOACs were at least as effective as warfarin in preventing stroke and systemic embolic events in patients with AF and were associated with lower rates of hemorrhagic stroke compared with warfarin [5–8]. Further, the NOACs were associated with similar or lower rates of major or clinically relevant nonmajor bleeding and significantly decreased rates of intracranial bleeding compared with warfarin. For the treatment of venous thromboembolism, the NOACs were noninferior to standard therapy (including subcutaneous enoxaparin or heparin followed by warfarin or acenocoumarol), decreased major bleeding risk, and decreased or produced similar clinically relevant nonmajor bleeding risk [9–14].

In a meta-analysis of the NOAC phase 3 trials for stroke prevention in AF, the NOACs reduced the risk of stroke or systemic embolic events by 19 % (relative risk ratio 0.81; 95 % confidence interval 0.73–0.91, *P* < 0.0001), primarily through reductions in the risk of hemorrhagic stroke and reduced rates of all-cause mortality and intracranial hemorrhage relative to warfarin; however, the risk of gastrointestinal bleeding was increased [15]. Despite initial reports of a higher prevalence of dabigatran-related bleeding, analyses of postmarketing data show similar or lower rates of bleeding compared with warfarin, as reported in phase 3 testing [16, 17]. Similarly, postmarketing rates of rivaroxaban-associated bleeding were consistent with phase 3 trial results [18]. Postmarketing results for apixaban and edoxaban have not yet been published.

Although NOACs reduce the risk of bleeding relative to warfarin, concerns remain regarding the management of bleeding related to NOAC use. Bleeding is a complication for all anticoagulants and concerns regarding bleeding risk may be a barrier to their prescription despite the known benefits of anticoagulation [19]. Additionally, concomitant medications such as antiplatelet therapy also increase the bleeding risk for both vitamin K antagonists (VKAs) and NOACs [20–22]. With short half-lives ranging from 5 to 17 h [1–4] and pharmacologic activity paralleling plasma concentrations, discontinuation of a NOAC will result in a relatively rapid return to normal hemostatic function compared with VKAs, as the NOACs are direct inhibitors of hemostatic factors. However, no agent has yet been approved for rapid reversal of anti-FXa NOAC anticoagulation, although one agent is available for urgent reversal of dabigatran-associated emergent bleeding [23]. Additionally, no single laboratory test has been established as the validated, universal measure of the NOACs’ anticoagulation effects, especially for FXa inhibitors. This review discusses the laboratory tests available for assessing anticoagulation, human models of bleeding, and the use of strategies—with a focus on prothrombin complex concentrates (PCCs)—for the reversal of anticoagulation by NOACs.

## Assessment of anticoagulation levels

Unlike VKAs, NOACs do not require routine coagulation monitoring, in part due to predictable pharmacokinetics and pharmacodynamics and fewer drug–drug and drug–food interactions [24]. Prothrombin time (PT) and international normalized ratio (INR) are routinely used to measure VKA anticoagulation levels but INR is not considered an accurate measure of anticoagulation in patients on NOACs. Unlike VKAs, PT/INR correction for NOACs does not eliminate the variability arising from use of different thromboplastins; this results in difficulty in standardizing clotting assays for clinical testing [25, 26].

In the event of emergent bleeding, determination of a patient’s level of anticoagulation and the ability to measure the effectiveness of a reversal agent are important. In addition to determining the management of bleeding, assessing the presence or absence of anticoagulation can also eliminate unnecessary and costly actions in attempting NOAC reversal. Thus, understanding the impact of NOACs on common laboratory coagulation tests and the relationship of this impact on clinical coagulation status is critical. To date, no single routine laboratory test has been identified to accurately measure the clinical anticoagulation state of patients on NOACs [27], although a number of assays are responsive to NOACs [25, 28, 29] (Table 1).

**Table 1:** Laboratory assays responsive to NOACs [25, 28, 29]

	Dabigatran	Rivaroxaban	Apixaban	Edoxaban	Issues related to testing
PT	Insensitive at therapeutic concentrations	Normal PT can exclude significant drug levels	Insensitive	Poor sensitivity	Highly variable based on reagent; cannot be standardized across laboratories
aPTT	Normal aPTT can exclude anticoagulation	Insensitive	Insensitive	Dose-dependent prolongation	Highly variable based on reagent; cannot be standardized across laboratories Clinical relevance of recovery unknown
TT	Highly sensitive, must be diluted Normal values can exclude anticoagulation	Insensitive	Insensitive	Insensitive	Preferred assay for dabigatran; reagent-dependent
Anti-FXa	Insensitive	Sensitive when calibrated; normal FXa can exclude anticoagulation	Sensitive when calibrated; normal FXa can exclude anticoagulation	Sensitive when calibrated; normal FXa can exclude anticoagulation	Variable range, interlaboratory variability, not widely available. Preferred assay for rivaroxaban, apixaban, and edoxaban

*aPTT* activated partial thromboplastin time, *FXa* direct factor Xa, *PT* prothrombin time, *TT* thrombin time

Activated partial thromboplastin time (aPTT) can provide an approximation of the anticoagulation effect of dabigatran and is a reasonable screening test for the presence of the direct thrombin inhibitor; however, variability in results should be expected and no guidance is provided regarding the clinical relevance of a particular level of recovery [1]. More sensitive assays for monitoring dabigatran include thrombin time (TT), diluted thrombin time, and ecarin clotting time (ECT) for determining specific levels of anticoagulation [1].

At therapeutic doses, PT and aPTT are prolonged by rivaroxaban, apixaban, and edoxaban. However, because these changes are quite small and variable and dependent on the thromboplastin employed [28–30], they are not recommended for routine monitoring of anticoagulant effects [2–4]. PT and aPTT are less sensitive to apixaban than to rivaroxaban and dabigatran in head-to-head comparison in platelet-poor plasma [31].

The anti-FXa assay—which assesses ex vivo inhibition of exogenously added factor Xa—shows a linear correlation with rivaroxaban, apixaban, and edoxaban concentrations and may provide a quantitative or semiquantitative measurement when conducted with a NOAC-specific calibrator [32–35]. Several chromogenic anti-FXa assays demonstrate a concentration-dependent increase in response to rivaroxaban, apixaban, and edoxaban [27, 28, 30, 32, 36, 37]. The anti-FXa reagents vary in dynamic range and there is significant interlaboratory variability for in vitro assessments [26, 33, 34]. Intrinsic FX activity can be assayed using a chromogenic method and factor X is activated by Russell’s viper venom in the presence of calcium but few data are available on the clinical utility of this assay [35].

Thrombin generation (TG), as measured by endogenous thrombin potential (ETP), is sensitive to NOAC-related anticoagulation [38–40]; however, this is an experimental assay that has not been approved for routine clinical use or been validated in a clinical setting. Sample collection and preparation can significantly affect the results of TG assays, making them challenging to implement. Although several commercial tests for ETP are available, the lack of standardization within and between clinical laboratories remains a barrier to greater inclusion in clinical practice [41].

Thromboelastography/rotational thromboelastography (TEG/ROTEM)-based assays can be affected by dabigatran, rivaroxaban, and apixaban [42, 43]. However, the results are variable and do not show dose-dependent responses at clinically relevant concentrations for rivaroxaban and apixaban [43–46]. The TEG test has been shown to be sensitive to dabigatran-spiked samples in vitro [47] and has shown a concentration-dependent response to dabigatran that correlates with ECT and Hemoclot results [48].

## Models of bleeding

Regardless of assay availability, no published studies suggest laboratory tests of hemostasis are predictors of bleeding risk for NOACs. Thus far, it has been difficult to establish a reliable human bleeding model to test novel inhibitors of the coagulation cascade. Reported models for measuring the effects of antiplatelet agents are limited by high variability and are not reliably predictive of individual responses to reversal agents or bleeding tendency in clinical settings.

Surgicutt (International Technidyne Corporation, Edison, NJ, USA) and Simplate (Organon Teknika, Durham, NC, USA) are disposable devices developed for clinical measurement of bleeding times from standardized incisions [49, 50]. The Surgicutt model is more sensitive to bleeding time compared with Simplate [49]. However, the Surgicutt model is associated with high intrasubject variability and limited reproducibility based on a high degree of interobserver variability [50]. Additionally, this method is relatively insensitive to the effects of VKAs or the NOACs on clotting time because the clots formed are highly platelet-dependent (so-called “white clots”) [21, 51, 52]. The use of bleeding times to routinely assess platelet function has been replaced with more sensitive assays using flow cytometry and specific biomarkers [53].

A limited number of human studies have evaluated anticoagulant-induced bleeding following punch biopsy. This method represents a more invasive, and perhaps less platelet-dependent, model of bleeding and is more clinically relevant to vasculature injury as encountered in surgery (or potentially in other wounds) relative to standardized incisions. This model was initially developed to detect the effects of warfarin in clinical bleeding assessments [54] and has subsequently been shown to be sensitive to the effects of clopidogrel and edoxaban [40, 55]. The punch biopsy is typically performed on the back of the leg and removes a uniform cylindrical core of cutaneous tissue 5 mm in diameter and 4 to 6 mm deep. Bleeding duration (BD) and bleeding volume (BV), measured by converting the weight of blood absorbed on pre-weighed filter paper disks, may be evaluated [40, 54, 55]. In healthy subjects, VKA anticoagulation increased BV and prolonged BD [54]. Administration of recombinant factor VIIa (rFVIIa) failed to reverse the VKA-induced increase in BV and prolongation of BD. Further, intersubject variability was noted in both measures at baseline [54]. The model was again applied in an assessment of the reversal of clopidogrel by rFVIIa. Clopidogrel prolonged BD and increased BV in healthy volunteers and rFVIIa reduced the clopidogrel-induced increases in BV to a greater extent than it reduced BD [55]. However, variability in baseline BD and BV was again noted and attributed in part to differences due to inter investigator performance of the procedure [55].

More recently, edoxaban reversal was evaluated using a rigorously standardized application of the punch biopsy method in a phase 1, double-blind, randomized, placebo-controlled two-way crossover study [40]. Investigators at the study center were extensively trained on the punch biopsy procedure to reduce variability in performing the procedure. The number of physicians performing the biopsy and assessing BD was limited; for most subjects, a single physician performed the punch biopsies at both visits. The efforts to standardize the punch biopsy method resulted in improved coefficients of variation for both BD and BV in relation to previous studies [40, 54, 55]. The intrasubject variability associated with BD (35 % and 26 % for edoxaban 60 mg and 180 mg, respectively) was lower than that associated with BV (37.5 % and 35.7 % for edoxaban 60 mg and 180 mg, respectively) [40]. Correlations between BD and BV with markers of anticoagulation were also assessed. The lag in TG was significantly correlated with BD (*P* = 0.04) and a trend toward correlation between ETP and BD was also reported (*P* = 0.07) [40]. Similarly, a trend toward a significant correlation between BD and PT and aPTT was also noted (*P* < 0.1). There was no correlation between anti-FXa or intrinsic FX activity and BD [40]. The reason for this lack of correlation is unclear but may reflect the fact that only two doses of edoxaban were studied and correlation was assessed at a single timepoint. Thus, the range in exposure may not have been large enough to establish a clear correlation. Taken together, these results suggested that both BD and BV were sensitive to edoxaban dosing and had acceptable variability and that TG and possibly PT would be appropriate biomarkers for future studies assessing reversal.

## Treatment of hemorrhage

Multiple definitions of bleeding based on different criteria are used in the literature. Generally, minor or mild bleeding does not pose much risk to the patient. Clinically relevant major or nonmajor bleeding events require medical intervention. Major bleeding is defined by the International Society on Thrombosis and Haemostasis as fatal bleeding; symptomatic bleeding in a critical area or organ; and/or bleeding resulting in a fall in hemoglobin of at least 2 g/dL or bleeding leading to transfusion of 2 or more units of whole blood or blood cells [56]. The Thrombolysis in Myocardial Infarction Study group definition of major bleeding includes any intracranial bleeding (excluding microhemorrhages <10 mm), clinically overt signs of hemorrhage with a drop in hemoglobin ≥5 g/dL, and/or fatal bleeding that directly results in death within 7 days; minor bleeding is defined as any clinically overt bleeding with a drop in hemoglobin of 3 to <5 g/dL [57, 58].

Reversal of NOACs is only necessary in cases of life-threatening bleeding or emergency surgery. In instances of minor bleeding, symptomatic management, such as nasal packing in the case of epistaxis, has been suggested for patients receiving NOACs [59]. If necessary, delaying the next dose of anticoagulant or temporarily discontinuing treatment—as the NOACs have relatively short half-lives—may be sufficient [1–4, 24, 59]. Administering activated charcoal to reduce NOAC absorption has also been suggested [24]. In instances of moderate or severe bleeding, recommendations are primarily supportive and are similar to those given for VKA-related bleeds; hemodynamic support to maintain blood pressure, renal perfusion, and urine output should be provided [60]. Maintenance of hemodynamic support and renal perfusion is important as 80 % of the clearance of an absorbed dabigatran dose [1] and 66, 50, and 27 % of the total clearance of rivaroxaban, edoxaban, and apixaban, respectively, is via the kidneys [2–4]. In the event of moderate-to-severe bleeding, the European Society of Cardiology guidelines recommend mechanical compression and volume replacement when appropriate [61]. Fresh frozen plasma, platelets, red blood cells, and cryoprecipitate (or fibrinogen concentrates if cryoprecipitate is not available) are important for hemostatic and hemodynamic resuscitation [62, 63]. Notably, the European Heart Rhythm Association (EHRA) does not recommend fresh frozen plasma in patients with life-threatening NOAC-related bleeding and instead suggests PCCs or factor VIII inhibitor bypassing agent (FEIBA, Baxter, Westlake Village, CA, USA), if available [64]. However, if the patient is actively hemorrhaging, then a massive transfusion protocol should be considered and instituted [64].

Fibrinolysis plays a major role in bleeding and coagulopathy and the use of antifibrinolytics such as tranexamic acid can be important additional adjunctive treatment strategies for major bleeding [65]. Tranexamic acid interferes with the binding of plasmin to fibrin and is used to treat bleeding and reduce the need for transfusion in cardiac surgery or trauma [60, 66, 67]. European Heart Rhythm Association guidelines suggest tranexamic acid as an adjuvant for non-life-threatening NOAC-related bleeding based on its use in other bleeding scenarios; however, animal data suggest that, by itself, tranexamic acid does not affect NOAC anticoagulation [68] and clinical data supporting its efficacy in reversal of NOAC anticoagulation are scarce [64]. Although tranexamic acid is not expected to reverse anticoagulation due to edoxaban [4] or other factor Xa inhibitors, it is an important multimodal consideration for repletion in critically ill and bleeding patients along with fibrinogen measurements [69].

## Prothrombin complex concentrates

**Fig. 1:**
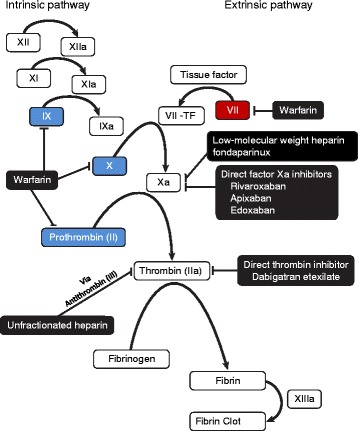
Sites of action of prothrombin complex concentrates, nonvitamin K antagonist oral anticoagulants, and warfarin on the coagulation cascade. *Blue boxes* indicate factors that are present in 3 F- and 4 F-PCCs; the red box indicates factor present in 4 F-PCCs. *3 F-PCC* 3-factor prothrombin complex concentrate, *4 F-PCC* 4-factor prothrombin complex concentrate

Prothrombin complex concentrates are pooled plasma products that contain varying amounts of inactive vitamin K-dependent clotting factors in addition to vitamin K-dependent proteins. The 3-factor PCCs (3 F-PCCs) include factors II, IX, and X; 4-factor PCCs (4 F-PCCs) include factors II, IX, VII, and X (Table 2) [70]. In addition, different PCC preparations contain varying levels of heparin and/or anticoagulant proteins (e.g., proteins C and S) to reduce their prothrombotic effects [70]. Both 3 F- and 4 F-PCCs replenish factors in the intrinsic and extrinsic coagulation cascade (Fig. 1). The 3 F-PCCs are an approved treatment for factor IX deficiency in patients with hemophilia B [71]; their use in anticoagulant reversal is an off-label application in the US and, until recently, only 3 F-PCCs were available in the US. A 4 F-PCC was recently approved in the US for the reversal of VKA anticoagulation in patients with acute major bleeding [72]. In the European Union, both 3 F- and 4 F-PCCs are available and 4 F-PCCs are approved for the reversal of VKAs [73].

**Table 2:** Variations in PCC composition^a^

	3 F-PCC		4 F-PCC					
	Bebulin® (Baxter)	Preconativ (Kabi)	Proplex-T^b^ (Baxter)	Beriplex P/N® (CSL Behring)	Kaskadil® (LFFB^c^)	Octaplex® (Octapharma)	Cofact® (Sanquin)	PPSB-HT Nichiyaku (Nihon)
Factor II	24–38	50	50	31	37	10–40	14–35	20
Factor VII	<5	NA	400	16	10	10–40	7–20	20
Factor IX	24–38	60	100	29	25	20–31	25	20
Factor X	24–38	50	50	41	40	10–40	14–35	20
Protein C				35		10–40		15
Protein S				25		10–40		
Protein Z				36				
Antithrombin III				0.6			<0.6	
Heparin	<0.15 IU/IU FIX		<1.5	0.5	5	4–15	None	

Table reproduced with permission from Bershad and Suarez [70]

*3 F-PCC* 3-factor PCC, *4 F-PCC* 4-factor PCC, *FIX* factor IX, *IU* international units, *NA* not applicable, *PCC* prothrombin complex concentrate

^a^Composition in international units/ml (IU/ml)

^b^Concentration not specified

^c^LFFB Laboratoire Français du Fractionnement et des Biotechnologies, France

### PCCs for NOAC reversal

Support for the reversal of the anticoagulant effect of NOACs in humans with PCCs is based primarily on case reports, evaluation of ex vivo parameters, and surrogate biomarker endpoints in healthy volunteers [74]. Thus far, only one published study has used a bleeding endpoint to assess PCC reversal [40]. At least one registry trial is underway to assess outcomes in bleeding patients or those requiring urgent care treated with NOACs and reversed with PCCs, rFVIIa, and/or hemodialysis, so these data may be available in the future [75].

Dabigatran, a direct thrombin inhibitor, acts downstream of the factors replaced by PCCs (Fig. 1). Previous studies reported that, by driving TG, PCC administration could potentially reverse dabigatran anticoagulation. As reported in ex vivo studies in plasma from dabigatran-treated patients, 4 F-PCC and activated PCC (aka FEIBA) reversed ETP to near-baseline values at low doses and dose-dependently increased TG, even at low doses [76, 77]. However, in vitro or in healthy subjects, as assessed by prolonged aPTT, ETP lag time, TT, ECT, and clotting time, a single bolus of 40 or 50 IU/kg 4 F-PCC was ineffective in reversal of dabigatran 150 mg twice daily [38, 78]. The use of FEIBA partially reduced clotting time in vitro [78] and reports suggest that the concentration of tissue factor present is a key element in the reversal of dabigatran [79].

Replenishing coagulation factors with 3 F- and 4 F-PCCs is more likely to affect the reversal of the FXa inhibitors than dabigatran. In vitro, apixaban prolongs TG lag time and reduces peak TG; both rFVIIa and FEIBA partially restored the prolonged TG lag and significantly enhanced peak TG. Similarly, 4 F-PCC partially recovered the TG lag but the effect did not reach significance [80]. At supratherapeutic concentrations of apixaban, 4 F-PCC partially restored ETP and peak TG and shortened PT [81]. However, TG lag time was unchanged by 4 F-PCC. rFVIIa had no effect on ETP but it did reduce TG lag time. FEIBA produced an overcorrection of ETP, shortened the apixaban-prolonged PT, increased peak TG, and decreased but did not fully correct TG lag time or aPTT [81]. In vivo, 4 F-PCC reverses the apixaban-prolonged PT and increases ETP at 15 minutes following infusion [82]. Between 6 and 24 h following PCC infusion, ETP returned to and increased above pre-apixaban levels [82].

Both 3 F- and 4 F-PCCs have also been shown to affect rivaroxaban TG parameters. Ex vivo, 4 F-PCC corrected ETP and peak TG modestly; rFVIIa was less effective in correcting ETP but it did revert rivaroxaban-induced lag time to baseline levels [76]. In contrast, FEIBA corrected ETP and peak thrombin as well as kinetic parameters of TG. However, most doses of PCC and FEIBA tested elevated TG over baseline values [76]. In vitro, both FEIBA and rFVIIa reversed rivaroxaban-induced prolonged PT and TG lag time and increased clotting time by roughly 50 % [83]. Peak thrombin was most strongly reversed by FEIBA [83]. Reversal of ETP by FEIBA or 4 F-PCC was dependent on rivaroxaban concentration; FEIBA was more effective in reversal than 4 F-PCC [83]. The use of rFVIIa produced only a partial reversal and TG was again elevated above baseline values [83]. In vivo, both 3 F-PCC and 4 F-PCC normalized ETP and PT [38, 39], with faster reversal of prolonged ETP by 3 F-PCC relative to 4 F-PCC [39]. The reduction of mean PT was greater with 4 F-PCC relative to 3 F-PCC but 3 F-PCC reversed TG to a greater degree than 4 F-PCC [39]. It should be noted that these studies highlight the contribution of different formulations of PCCs as the PT reduction can differ in magnitude based upon the 4 F-PCC chosen [38, 39]. This is attributed to the varying concentrations of heparin and anticoagulatory proteins included in different preparations [39]. Both 3 F- and 4 F-PCC initially prolonged aPTT, which gradually returned to baseline values [39]. No thromboembolic events occurred in these studies [38, 39].

In pooled plasma, a 1000-ng/mL dose of rFVIIa reversed the prolonged PT induced by edoxaban 150 ng/mL or 300 ng/mL to near control values. Both FEIBA and 4 F-PCC also significantly reduced PT but did not correct PT to baseline [84]. Ex vivo, both FEIBA and rFVIIa partially reversed edoxaban-mediated changes to PT, aPTT, and anti-FXa at supratherapeutic concentrations of edoxaban [85]. Neither rFVIIa nor FEIBA significantly reversed the anticoagulatory effects of edoxaban as measured by intrinsic FX activity. Further, no dose response was observed for rFVIIA or aPCC [85].

In a phase 1 clinical trial, 3 F-PCC was shown to completely reverse the effects of edoxaban on ETP but failed to reverse edoxaban-induced prolongation of PT [86]. A transient dose-dependent increase in prothrombin fragment 1 + 2 was observed, suggesting a possible procoagulant effect, although no thromboembolic events occurred [86]. However, using the above-described punch biopsy bleeding model in healthy subjects, 4 F-PCC produced a dose-dependent reversal of the effects of edoxaban on both BD and ETP [40]. A 50-IU/kg dose of 4 F-PCC completely reversed ETP to baseline values [40]. A similar reduction in BV was noted, while PT was only partially reversed [40]. Again, no thromboembolic events occurred [40]. To date, this is the only study to demonstrate NOAC reversal with a 4 F-PCC with results correlating to a bleeding endpoint. Further, this study suggests that ETP may represent an important biomarker for edoxaban-related anticoagulation effects [40].

There is a lack of good correlation between PT and aPTT prolongation and the anticoagulant activity of FXa inhibitors and, hence, reversal, despite the wide availability and ease of these tests. Furthermore, TG (specifically ETP) may better correlate with anticoagulant activity and reversal but is an impractical method, particularly in emergency situations, due to central laboratory capabilities needed for everyday testing. Anti-FXa assays are in development and correlate best with FXa inhibitor activity and potentially reversal with specific agents but are not appropriate for measurement of reversal by PCCs.

## Other reversal agents

The dabigatran-specific humanized monoclonal antibody fragment idarucizumab was recently approved for dabigatran-treated patients when reversal of anticoagulant effects is needed for emergency surgery or urgent procedures or in cases of life-threatening or uncontrolled bleeding [23]. In an interim analysis of a prospective cohort study, 5 g of idarucizumab rapidly reversed the anticoagulant effects of dabigatran in patients who required surgery or experienced life-threatening bleeding, with no procoagulant effect observed after administration [87]. Among patients with elevated ECT and dilute TT at baseline, idarucizumab rapidly normalizes the anticoagulant activity of dabigatran in 88 to 98 % of the patients, with only one thrombotic event reported within 72 h of idarucizumab administration [87]. Following reversal of the anticoagulant effect, reintroduction of dabigatran may be initiated as early as 24 h after idarucizumab treatment [88].

Other agents are under investigation. Andexanet (andexanet alfa, Portola Pharmaceuticals, South San Francisco, CA, USA), a specific reversal biological agent that binds to FXa inhibitors and neutralizes the anticoagulant effects of both direct and indirect FXa inhibitors, has demonstrated efficacy in preclinical studies and in reversal of the effects of NOACs on anti-FXa and TG in clinical studies [89, 90]. Andexanet alfa has been submitted to the US Food and Drug Administration for approval [91]. In the ANNEXA-A and ANNEXA-R (Andexanet Alfa, a Novel Antidote to the Anticoagulation Effects of FXA Inhibitors Apixaban [ANNEXA-A] and Rivaroxaban [ANNEXA-R]) randomized, double-blind, placebo-controlled studies evaluating the ability of andexanet to reverse anticoagulation with apixaban and rivaroxaban in elderly healthy volunteers, reversal of the anticoagulant activity of apixaban and rivaroxaban (as measured by the anti-FXa assay) occurred within 2 to 5 minutes after administration of a bolus of andexanet. This effect persisted for 2 h following administration of a bolus of andexanet and for 1 to 2 h following administration of andexanet as a bolus plus a 2-h infusion, without evidence of serious adverse events or clinical thrombosis [90]. It is uncertain, however, whether 2 h of reversal is sufficient for patients at risk of factor Xa inhibitor-associated acute major bleeding. A longer infusion time may be required for NOAC patients who require emergency surgery or continue to bleed. Furthermore, the use of andexanet in combination with tranexamic acid, factor concentrates, or PCCs has not yet been investigated. A clinical trial to evaluate the efficacy and safety of andexanet in patients with FXa inhibitor-associated acute major bleeding is ongoing (ClinicalTrials.gov number NCT02329327). This study will not include patients requiring emergency surgery or procedural interventions.

Finally, the synthetic small molecule antidote ciraparantag (PER977, Perosphere, Danbury, CT, US) is effective in reversal of all four NOACs as assessed by a rat tail transection bleeding model as well as in reversal of edoxaban based on TEG measurements [92, 93]. Ciraparantag has also been shown to reverse the effects of a single dose of edoxaban on whole blood clotting time and restore hemostasis in healthy subjects [94]. A clinical trial to investigate the safety, tolerability, and effect of ciraparantag following re-anticoagulation with edoxaban and its ability to re-reverse edoxaban is ongoing (ClinicalTrials.gov number NCT02207257).

## Conclusions

The PCCs are important therapeutic approaches for hemostatic resuscitation and in surgery and general trauma. They are widely available for reversal of bleeding associated with VKA anticoagulation and show promise for reversal of NOAC-associated bleeding. The widespread acceptance of PCCs for NOAC reversal will be aided by the development of standardized assays for NOAC anticoagulation and reversal. However, in a life-threatening hemorrhage, basic resuscitative principles should be considered, with hemodynamic and hemostatic resuscitation of patients. The role of PCCs and other adjunct factors are important multimodal factors for therapy. Point-of-care anti-FXa tests may be useful tools to develop for assessing anticoagulation status and specific reversal agent effects but will not be appropriate for the measurement of reversal by PCCs.

## Abbreviations

3 F, 3-factor; 4 F, 4-factor; AF, atrial fibrillation; aPTT, activated partial thromboplastin time; BD, bleeding duration; BV, bleeding volume; ECT, ecarin clotting time; ETP, endogenous thrombin potential; FEIBA, factor VIII inhibitor bypassing agent; FXa, direct factor Xa; INR, international normalized ratio; NOAC, novel oral anticoagulant; PCC, prothrombin complex concentrate; PT, prothrombin time; rFVIIa, recombinant factor VIIa; TEG, thromboelastography; TG, thrombin generation; TT, thrombin time; VKA, vitamin K antagonist
